# Evaluation of the success rate of various inpatient alcohol addiction treatment programs in the Czech Republic and their comparison

**DOI:** 10.1192/j.eurpsy.2023.1417

**Published:** 2023-07-19

**Authors:** T. Skorkovsky, J. Vevera, M. Benis, M. Miovsky, P. Popov

**Affiliations:** 1 Charles University, Faculty of Medicine in Pilsen; 2 University Hospital in Pilsen, Pilsen; 3 Charles University, 1st Faculty of Medicine; 4 General University Hospital in Prague, Prague, Czech Republic

## Abstract

**Introduction:**

Harmful alcohol use far exceeds other mental disorders in the proportion of patients who do not receive adequate treatment. Despite the long history of anti-alcohol treatment in Czech Republic, there is no published prospective study to this day, in which patients that underwent inpatient addiction treatment are compared to patients on the waiting list and only one prospective randomized study comparing two different medium-term inpatient programs was published.

Almost all the studies published so far only bring results of particular hospitals. Differences in methodology, differences between cohorts of patients, absence of profiling and differences in therapeutic programs and historical changes makes comparison of results of those studies very difficult.

**Objectives:**

This work seeks to present and compare the data from studies that evaluate the success of medium-term inpatient treatment of alcohol dependent patients in the Czech Republic. Another aim was to identify problems that make such comparison difficult.

**Methods:**

Bibliographia Medica Čechoslovaca and Pubmed was used to find studies published in professional medical journals since 1970, in which abstinence of patients who voluntarily completed medium-term inpatient treatment of alcohol dependence is evaluated.

**Results:**

Medium-term inpatient treatment of alcohol addiction leads to one year abstinence in 34 to 76 % of patients. Such variance value is largely caused by different methodology of compared studies. In compared studies there are differences:

1. in definition of abstinence

2. if abstinence rate is assessed in all patients who have entered the treatment or only in those who have completed the treatment properly

3. if abstinence rate is calculated using number of patients entering treatment or the number of patients who have been obtained by valid information (outpatient clinic, questionnaires)

4. in the way the data was collected

5. in the composition of patients

6. in societal background, because there is large time gap present between compared studies

**Conclusions:**

The comparison of individual studies presented many problems. Further steps should be taken to help compare treatment programs in the future, as they provide different therapeutic interventions in different intensity and length to different patients. Adequate patient profiling, detailed description of therapeutic interventions and identification of effective components of the therapeutic program is a way to support further research in this area, optimize existing programs and increase the overall efficiency of treatment.

**Disclosure of Interest:**

None Declared

